# A Rare Presentation of Sphenoid Sinus Lymphoma in an 18-Year-Old Female

**DOI:** 10.7759/cureus.97534

**Published:** 2025-11-23

**Authors:** Noor Binsanad, Latifa Fakhroo, Abdulla Darwish, Bedoor Alomran, Mohammed Albalooshi

**Affiliations:** 1 Otolaryngology, Bahrain Defence Force Royal Medical Services, Riffa, BHR; 2 Diagnostic Radiology, Bahrain Defence Force Royal Medical Services, Riffa, BHR; 3 Pathology and Laboratory Medicine, Bahrain Defence Force Royal Medical Services, Riffa, BHR; 4 Neurological Surgery, Bahrain Defence Force Royal Medical Services, Riffa, BHR

**Keywords:** cranial nerve involvement, cranial nerve palsy, diplopia, lymphoma, paranasal sinus diseases, paranasal sinus neoplasm, pre-b lymphoblastic lymphoma, sphenoid sinus lesions, sphenoid sinus tumor

## Abstract

Sphenoid sinus lymphomas are extremely rare, constituting a small fraction of both sphenoid sinus malignancies and extranodal non-Hodgkin lymphomas. Their clinical presentation can vary widely, ranging from symptoms resembling chronic sinusitis to more severe manifestations such as nerve palsies and visual disturbances.

An 18-year-old female with no significant medical history presented with blurred vision, diplopia, and a left-sided headache lasting one week. Neurological examination revealed mild limitation of lateral rectus movement in the left eye. Imaging studies, including non-contrast CT and MRI, identified a hyperdense mass arising from the left sphenoid sinus. A biopsy obtained via a transnasal sphenoidal approach confirmed a diagnosis of pre-B cell lymphoblastic lymphoma. Following the biopsy, the patient was treated with IV dexamethasone, leading to complete resolution of her symptoms and regression of the mass on follow-up imaging. Staging confirmed the diagnosis of stage 1A B-cell lymphoblastic lymphoma. The patient underwent chemotherapy and radiotherapy and remains in remission five years later. However, she developed bilateral hip avascular necrosis as a side effect, requiring hip replacement surgery.

Diagnosing sphenoid sinus lymphoma can be challenging due to its nonspecific symptoms and the complex anatomy of the sphenoid sinus, often leading to misdiagnosis as more common conditions such as sinusitis or cranial nerve palsies. Imaging, particularly MRI and CT, plays a critical role in assessing the disease's extent and evaluating potential cranial nerve involvement. A biopsy is essential for obtaining a definitive histological diagnosis to guide appropriate treatment plans. While steroids can offer rapid symptom relief, the primary treatment for sphenoid sinus lymphoma involves chemotherapy, often in combination with radiotherapy. Surgery is typically not recommended due to the tumor’s location and potential risks to surrounding structures.

The case highlights the importance of considering neoplastic lesions in patients presenting with unexplained headaches and ocular findings to support timely diagnosis.

## Introduction

Sphenoid sinus lymphomas are a rare presentation of extranodal lymphomas. Only 10-34% of non-Hodgkin lymphomas arise from extranodal sites, with nasal and paranasal lymphomas accounting for less than 3% [1]. Malignancies of the sphenoid sinus in general are not common, accounting for less than 1% of all cancers [2], and most are often squamous cell carcinomas and adenocarcinomas. Primary lymphomas of paranasal sinuses constitute around 8% of all paranasal malignancies [3]. Clinical presentations vary widely, ranging from vague, sinusitis-like symptoms to acute manifestations such as cranial nerve palsies and visual disturbances.

## Case presentation

An 18-year-old female with no significant past medical history presented to the emergency department with a history of blurred vision and diplopia. Her symptoms started one week prior and were associated with a left-sided, intermittent headache, primarily at night, which was not relieved by paracetamol. She had no history of fever, weight loss, or night sweats. On examination, she had minimal tenderness over the left side of her head and left eye, with no enlarged peripheral lymph nodes. Chest and abdominal examinations were unremarkable. Ophthalmologic assessment revealed good visual acuity, normal intraocular pressure, and mild limitation of lateral rectus movement in the left eye with associated pain. The optic nerve appeared elevated with blurred margins, and papilledema could not be ruled out. Laboratory investigations, including complete blood count, renal and liver function tests, and C-reactive protein, were within normal limits.

**Figure 1:**
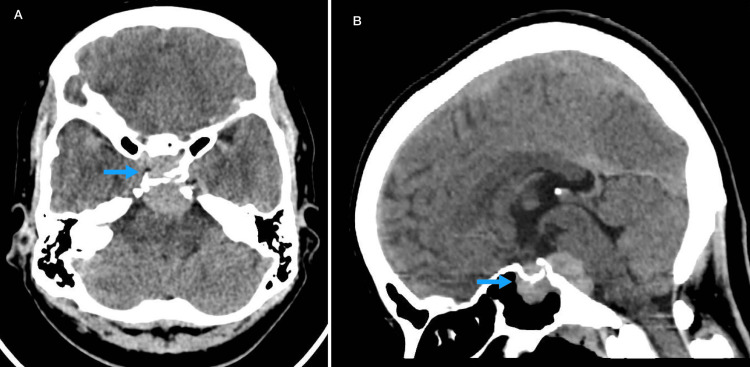
Computed tomography (CT) findings. (A) Axial non-contrast CT brain showing a hyperdense extra-axial mass arising from the sphenoid sinus (arrow). (B) Sagittal non-contrast CT brain showing the same hyperdense extra-axial mass arising from the sphenoid sinus (arrow).

**Figure 2:**
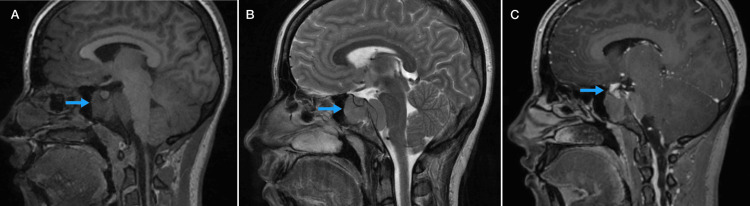
Magnetic resonance imaging (MRI) findings. (A) Sagittal T1-weighted MRI brain showing a homogeneous soft-tissue lesion arising from the sphenoid sinus with a hypo-intense signal (arrow). (B) Sagittal T2-weighted MRI brain showing a homogeneous soft-tissue lesion arising from the sphenoid sinus with a hypo-intense signal (arrow). (C) Sagittal T1-weighted post-contrast MRI brain demonstrating a soft-tissue lesion arising from the sphenoid sinus with diffuse restriction and mild enhancement (arrow).

Non-contrast CT of the brain revealed a hyperdense extra-axial mass arising from the left sphenoid sinus extending around the clivus and into the prepontine cistern. Minimal bone resorption was noted; however, there was no bony destruction or suprasellar extension (Figure 1). MRI confirmed a homogenous soft tissue lesion extending from the left sphenoid sinus to the sella turcica, clivus, and prepontine cistern. The lesion was hypointense on both T1 and T2 sequences (Figures 2A-2B), showed diffuse diffusion restriction, and demonstrated subtle post-contrast enhancement on T1 imaging (Figure 2C).

**Figure 3:**
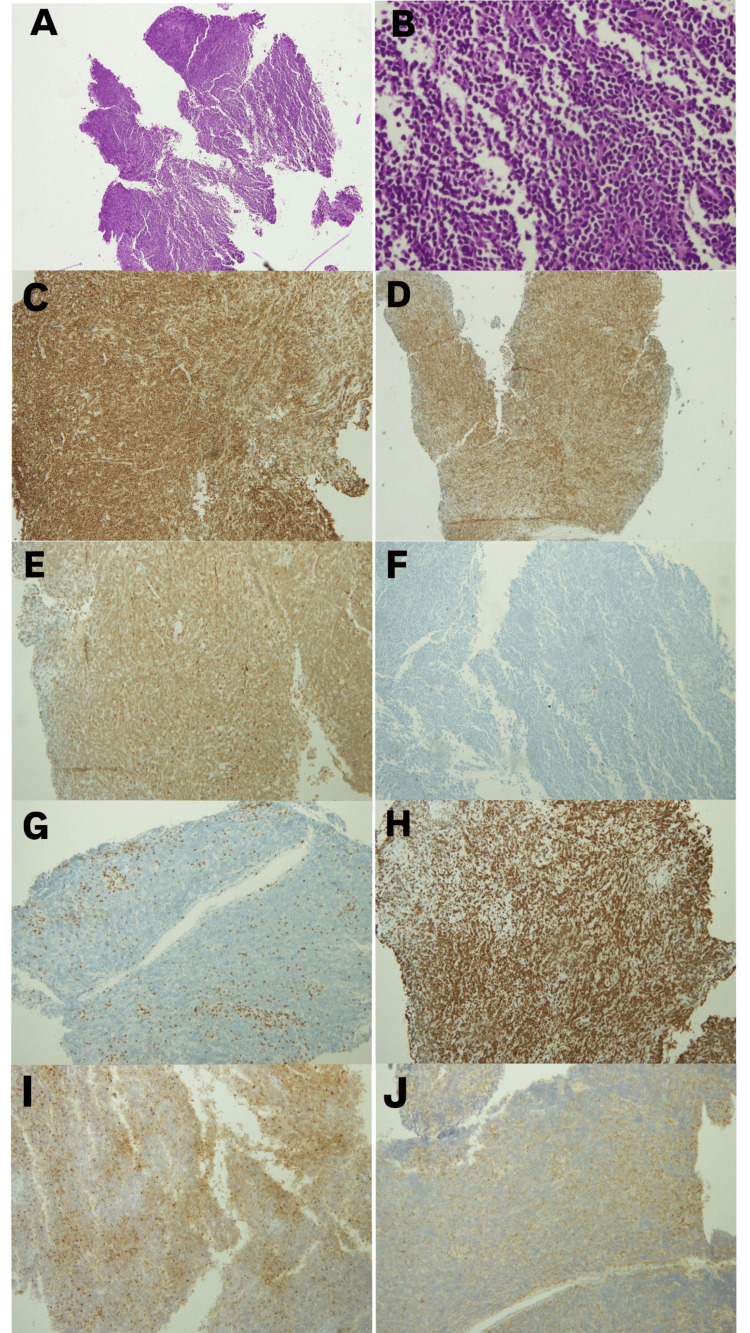
Histopathological examination, including immunohistochemistry using the Roche Ventana ultraView Universal DAB kit (Ventana Medical Systems, Inc., Tucson, AZ, USA), showing neoplastic lymphoid cells beneath the normal pseudostratified ciliated respiratory epithelium. (A) Infiltrative small blue cells on hematoxylin and eosin staining at low power (10×). (B) Infiltrative small blue cells on hematoxylin and eosin staining at high power (40×). (C) Positive for CD10. (D) Positive for Bcl2. (E) Positive for CD79a. (F) Negative for CD20. (G) Negative for CD3, with reactive T cells in the background. (H) Ki-67 proliferation index of 90%. (I) Positive for TdT. (J) Positive for CD99.

The patient underwent a transnasal sphenoidal approach for a tumour biopsy. The pathological examination concluded a diagnosis of B-cell lymphoblastic lymphoma. Multiple tissue sections were examined. The frozen section comprised diffuse infiltrative malignant cells, which displayed markedly pleomorphic nuclei with significant mitotic activity, some of them having abnormal forms. The paraffin sections were examined and showed diffuse sheets of medium-sized neoplastic lymphoid cells below the normal pseudostratified ciliated respiratory epithelium. The cells had irregular, round nuclei with focal cleaving. The tumor cells were strongly positive for CD10, Bcl2, and CD79a. They were moderately strongly positive for TdT and CD99 and showed weak focal positivity for cyclin D1 and CD56. They were negative for CD20, synaptophysin, chromogranin, CK7, desmin, S100, CD21 and CD15. CD3 was positive in the background reactive lymphocytes. Ki-67 showed a 90% proliferation index (Figure 3).

**Figure 4:**
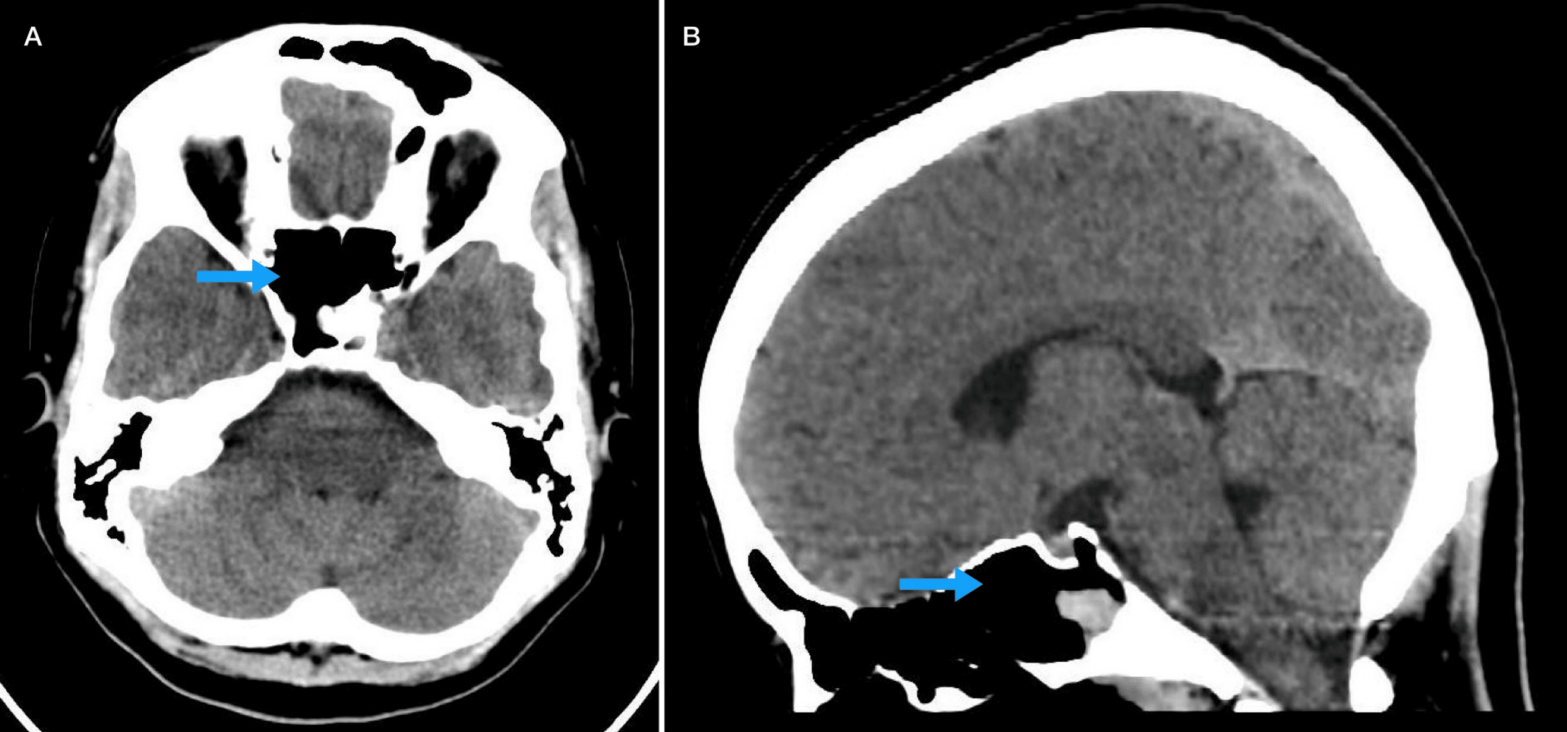
Follow-up CT brain findings. (A) Axial repeat non-contrast CT brain showing regression of the lesion (arrow). (B) Sagittal repeat non-contrast CT brain showing regression of the lesion (arrow).

Following the biopsy, the patient was started on IV dexamethasone, which was later transitioned to oral tablets upon discharge and tapered over a total of four weeks. On one-week follow-up, repeat CT imaging demonstrated significant regression of the lesion (Figure 4), and her headache and diplopia had resolved completely. She was subsequently referred to the oncology center for further staging and management. Further work-up confirmed a final diagnosis of stage 1a B-cell lymphoblastic lymphoma. She received the Dana Farber Protocol for Acute Lymphoblastic Leukaemia/Lymphoblastic Lymphoma, followed by BFM2000 1B Consolidation Protocol and Protocol M, as well as radiotherapy. She subsequently developed bilateral hip avascular necrosis as a treatment-related complication, for which she underwent bilateral total hip replacement. She has continued regular follow-up with serial positron emission tomography (PET) scans and MRI for five years, all of which have consistently shown no evidence of recurrent or residual disease, and she remains in complete remission.

## Discussion

Lymphomas of the sphenoid sinus are extremely rare. Sphenoid sinus malignancies represent <1% of all cancers [2]. Common presenting symptoms include, but are not limited to, headaches, diplopia, nausea, vomiting, and symptoms of sinusitis. The sphenoid sinus is closely associated with the optic canal, dura mater, pituitary gland, and cavernous sinus, which houses the internal carotid arteries, as well as the oculomotor (CN III), trochlear (CN IV), ophthalmic (CN V1), maxillary (CN V2) and abducens (CN VI) nerves, and because of its thin bony walls, tumor invasion or compression of these adjacent neurovascular structures can readily occur, explaining neurological manifestations such as cranial nerve palsies and diplopia [4]. A sphenoid sinus lymphoma presenting as an isolated nerve palsy without other neurological deficits is not common; however, there have been cases that did present with palsies of some of the previously mentioned nerves, most common being the abducens nerve (CN VI) [1]. Diplopia secondary to sixth nerve palsy is one of the earliest signs of sphenoid sinus disease [1]. Some cases may mimic Tolosa-Hunt syndrome, similar to our case, which presents with unilateral orbital pain/headache, and nerve palsies of cranial nerves III, IV, and VI [5]. The average age of the documented sphenoid sinus lymphoma cases has been noted to be between 50 and 60 years and with the majority being males [6]. A recent study found the male-to-female ratio was 2:1, consistent with prior studies suggesting a male predominance [5]. The average age of presentation for males was also lower than that of females by around six years [6]. With regard to laboratory investigations, several cases of paranasal sinus lymphoma have shown an association with Epstein-Barr virus (EBV), particularly cases of extranodal NK/T cell lymphomas [7,8]. Testing for EBV in biopsy samples can give insight into diagnosis and prognosis, and allow tailoring an appropriate treatment strategy [8]. Diagnostic imaging plays a crucial role in diagnosing and treating sphenoid lesions due to its unfavorable anatomical location. Thus, utilizing both CT and MRI scans together is essential for accurate diagnosis. CT imaging is significant in detecting bony erosions and destructions, while MRI is critical in delineating both intracranial and extracranial soft tissue invasion and assessing potential cranial nerve involvement [9]. In the present study, we performed a non-contrast CT scan, which showed a hyperdense extra-axial mass arising from the left sphenoid sinus extending around the clivus and into the prepontine cistern, with evidence of bony resorption. The MRI features revealed a homogenous soft tissue lesion with hypointense signal in T1 and T2, and showed diffusion restriction. This appearance was similar to previous published cases [10,11]. However, in our study, there was a slight enhancement post-contrast, compared to previous reports showing significant post-contrast enhancement, and others showing heterogeneous tumor appearance [1,12]. This could be explained by various factors influencing MRI characteristics, as intracranial heterogeneous lesions are usually caused by necrosis, hemorrhage, or varying cellularity within the tumor [13]. Clinically, the patient had diplopia and mild limitation of left lateral rectus movement, consistent with a left abducens nerve (cranial nerve VI) palsy, which aligns with the lesion’s extension into the prepontine cistern. Mild optic disc elevation likely reflects early mass effect on the optic nerve, and the patient’s headache is probably due to local compression from the sphenoid sinus and clival involvement. This case demonstrates how imaging characteristics of sphenoid sinus lymphoma can explain neurological symptoms and guide timely diagnosis and management.

Treatment of sphenoid sinus lymphoma requires a multidisciplinary approach. The primary treatment often includes combination chemotherapy and radiotherapy [5]. The most common chemotherapy regimen that has been used is the CHOP regimen (cyclophosphamide, doxorubicin, vincristine, and prednisolone), and it is frequently supplemented with rituximab, which was found to improve survival, especially in women [5,6]. Occasionally, BACOP (bleomycin, doxorubicin, cyclophosphamide, vincristine, and prednisolone) and CEOP (cyclophosphamide, epidoxorubicin, vincristine, and prednisolone) regimens are used [5]. Steroids can be helpful in the short term to manage acute symptoms when there is a need for rapid control before the definitive diagnosis and treatment, as in our case, where symptoms resolved after steroids prior to the final diagnosis. Surgery is considered only in select cases, such as a focal tumor with clear margins, rather than a primary treatment choice for all, due to the delicate location of the sphenoid sinus and the potential invasion to the surrounding tissues, as the majority of cases are not responsive to surgery [5]. There are no clear guidelines set for the optimal treatment of this entity of lymphomas; however, patients who are treated with combination chemo-radiation were found to have better overall survival than those who receive chemotherapy or radiation alone [14]. Endoscopic sinus surgery may offer a valuable approach for the prompt diagnosis of different sphenoid lesions. A balance needs to be made between acquiring sufficient tissue samples for diagnosis while minimizing the risk of injuring nearby critical structures, which may pose a significant challenge for surgeons. A key benefit of this surgical approach is that it allows direct access to the sphenoid sinus, enabling continuous monitoring of the tumor and its response to treatment through routine nasoendoscopy [15].

## Conclusions

Given the rarity of lymphomas arising in the paranasal sinuses and their variable presentations, this case highlights the importance of considering neoplastic causes in patients with unexplained headaches and ocular or sinus symptoms. Greater clinical awareness may help ensure timely diagnosis and management.
